# Phenological and Environmental Factors’ Impact on Secondary Metabolites in Medicinal Plant *Cotinus coggygria* Scop.

**DOI:** 10.3390/plants12091762

**Published:** 2023-04-25

**Authors:** Alexandra-Gabriela Ciocan, Victorița Tecuceanu, Cristian Enache-Preoteasa, Elena Monica Mitoi, Florența Elena Helepciuc, Tatiana Vassu Dimov, Alexandra Simon-Gruita, Gina Carmen Cogălniceanu

**Affiliations:** 1 Department of Developmental Biology, Institute of Biology Bucharest of Romanian Academy, 296 Splaiul Independenței Street, 060031 Bucharest, Romania; alexandra.ciocan@ibiol.ro (A.-G.C.); florenta.helepciuc@ibiol.ro (F.E.H.); gina.cogalniceanu@ibiol.ro (G.C.C.); 2 Faculty of Biology, University of Bucharest, Splaiul Independentei 91-95, 050095 Bucharest, Romania; vassut@yahoo.com (T.V.D.); alexandra.simon@bio.unibuc.ro (A.S.-G.); 3 “C.D. Nenitzescu” Institute of Organic and Supramolecular Chemistry, Romanian Academy, 202 B Spl. Independentei, 060023 Bucharest, Romania; 4 National Phytosanitary Laboratory, 11 Voluntari Boulevard, 077190 Voluntari, Romania

**Keywords:** *Cotinus coggygria*, secondary metabolism, environmental factors, phenological stages, polyphenols, flavonoids, LC–MS/MS, HPLC–UV–VIS–DAD, antioxidant capacity

## Abstract

*Cotinus coggygria* Scop. (smoketree) is a phytotherapeutically valuable shrub growing in specific areas in many Eurasian countries. Exploring the intrinsic and extrinsic (abiotic) factors that modulate its secondary metabolism has fundamental and applicative importance. Three smoketree plants from the same population were studied for a period of 4.5 months. Their extracts were characterized using LC–MS/MS, HPLC–UV–VIS–DAD and colorimetric assays to determine the chemical composition and antioxidant potential. Multivariate analysis was applied to correlate the metabolomic data with registered habitat variables and phenological stages. The identified and quantified compounds belonged to the flavonoids (myricetin-3-O-galactoside, myricitrin) and hydrolysable tannins groups (pentagalloyl glucose, methyl gallate, methyl digallate I). Phenolic compounds and tannins were synthesized abundantly in the flowering and fruit stages, whereas flavonoids and triterpenes accumulated during senescence. The antioxidant activities varied between detection methods, samplings and individuals and were only punctually correlated with the compound contents in certain phenological stages. Based on the HCAbp analysis, the samples clustered under four groups, according to their metabolic profile. The CCA analysis revealed that during the reproductive stages (flower, fruit or seed), the secondary metabolism of the plants’ leaves is sensitive to the action of abiotic factors, while in senescence, the metabolic content is according to the phenological phase. This study provides a first attempt at understanding the interplay between the habitat and the metabolome of smoketree.

## 1. Introduction

A plant’s primary metabolism consists of ubiquitously distributed compounds (carbohydrates, lipids and amino acids), directly involved in the growth and development of plants [1]. Derived from the primary metabolism [2], the secondary metabolism includes thousands of compounds belonging to one of the three major groups: terpenes, phenolic compounds and nitrogen-containing compounds [3]. Plants synthesize these molecules as a means of protection and adaptation against environmental factors, pathogens, insects and herbivores, but also to ensure one’s reproductive success, by attracting pollinators [4].

Numerous studies have reported the modulatory effect of environmental factors, such as humidity, temperature, precipitation and solar radiation, on the production of highly diverse secondary metabolites, in various plant species [1,2,3,4,5,6,7,8,9]. For example, in *Centella asiatica* (L.) Urban leaves, the content of terpene and polyphenolic compounds increased after exposure to sunlight for an entire day compared to plants exposed to 50% of light [10]. At the same time, temperatures of 40 °C caused a significant increase in alkaloids, such as vinblastine and catharanthine, in *Catharanthus roseus* (L.) G. Don seedlings as compared to exposure to 30 °C [11].

In the face of the ongoing climatic changes and the demands for natural alternatives to synthetic medicines, studies aiming to describe the secondary metabolism of plants in their natural environment, in correlation with developmental or phenological stages, are highly important. They could provide valuable information on the biology of the studied species, increase the chances of developing and applying efficient conservation strategies, and, finally, aid in discovering new medicinally and industrially valuable compounds. Although longitudinal studies exploring the impact of the environment on the secondary metabolism of species native to Central and South America exist [4,12,13,14,15,16,17,18], these aspects are poorly understood in species growing in a temperate climate.

*Cotinus coggygria* Scop., commonly known as smoketree, is a shrub belonging to the Anacardiaceae family, which reunites some economically important species, such as *Pistacia vera* L. (pistachio), *Mangifera indica* L. (mango), *Anacardium occidentale* L. (cashew tree) and *Rhus coriaria* L. (sumach) [19]. This species is easily recognizable by its characteristic pink pubescent floral peduncles (hence the name smoketree), which appear in the summer and the red hues of its foliage in fall [20,21,22]. Its presence is sporadic and discontinuous and has been signaled along the Mediterranean coast to the S-E of Europe (in 18 European states), but also in Turkey, Russia and many regions of China (Central, S-E and S-V) and northern India, which might suggest that its presence could depend on a series of environmental factors [23,24,25,26,27,28]. In the regions where it grows, several parts of the plant have been used by traditional medicine to treat skin [29,30], oral [31], respiratory [32], digestive [32], urinary [32] and cardiovascular diseases [32] and nowadays different types of cancer [22].

The complex secondary metabolism of smoketree was previously studied using different parts of the plant (heartwood, young shoot, twig, stem, leaf, inflorescence and fruit) and the detected compounds belonged to diverse phytochemical classes: (1) phenolic acids (gallic acid, chlorogenic acid, caffeic acid and rosmaric acid) [33,34,35]; (2) flavonoids (sulfuretin, cotinignan A, butein, taxifolin, fisetin, myricetin, kaempferol, apigenin, petunidin-3-glucoside, delphinidin-3-galactoside and cyanidin-3-galactoside) [22,33,34,35,36,37,38,39]; (3) hydrolysable tannins derived from gallic acid (pentagalloyl glucose, glucogallin and gallocatechin) [33,40] and (4) terpenes (limonene, (Z)-*β*-ocimene, myrcene, geranyl acetate, *β*-caryophyllene, sabinene, terpinolene and α-pinene), in variable quantities in respect to collection site, period and organ [23,25,26,41,42,43,44,45]. Several secondary metabolites identified in smoketree have been reported to present valuable medicinal applications. Myricetin-3-O-galactoside is a flavonoid that possesses antioxidant and antigenotoxic abilities [46] and has been proven to reduce nociception and inflammation in mice [47]. Myricitrin (myricetin-3-O-rhamnoside) has been described as an efficient antioxidant and antigenotoxic compound [46] and displayed promising antibiofilm formation [48], anti-influenza A virus [49], antihepatitis B virus [50] and wound-healing capacities [51]. Methyl gallate has shown protective effects against H_2_O_2_-induced oxidative stress in human umbilical vein endothelial cells [52] and against H_2_O_2_-induced apoptosis in PC12 cells [53]. Pentagalloyl glucose is a hydrolysable tannin that has displayed antiaging [54], antiobesity [55], antimicrobial, antidiabetic, anti-inflammatory and antitumor activities [56].

The present study aimed to characterize the secondary metabolism dynamic of *Cotinus coggygria* Scop. (leaves) and the antioxidant profile during the warm season, in Romania. This goal was achieved by (1) monitoring the total content of phenols, flavonoids, tannins and triterpenoids; (2) screening and quantifying the accumulation of five major compounds belonging to the hydrolysable tannins and flavonoids groups, through LC–MS/MS and HPLC–UV–VIS–DAD; (3) measuring the antioxidant activities and performing correlations with metabolites accumulation; and (4) integrating the metabolomic data with environmental variables and phenological stages. To the best of our knowledge, this is the first study to consider the impact of both extrinsic (environment) and intrinsic (phenological stages) factors on the secondary metabolism and antioxidant potential of smoketree.

## 2. Results

### 2.1. Total Content of Phenols (PC), Flavonoids (FC), Tannins (TC) and Triterpenoids (TTC)

**Figure 1:**
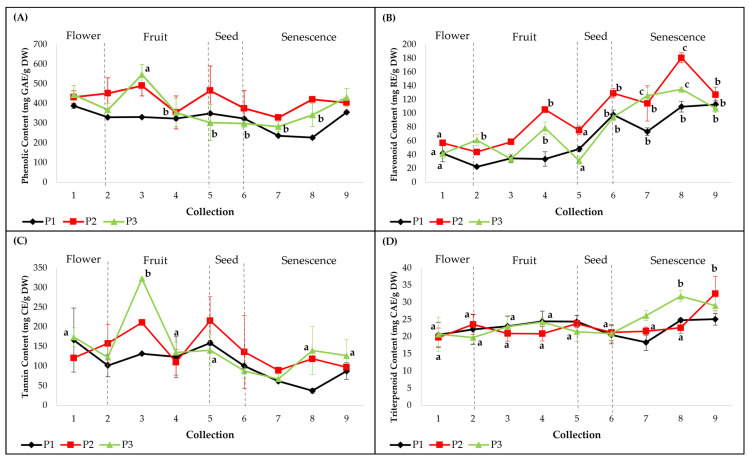
Quantitative determinations of total compound classes in smoketree methanolic extracts: (**A**) phenolic content (PC), (**B**) flavonoid content (FC), (**C**) tannin content (TC) and (**D**) triterpenoid content (TTC). P1 = plant 1; P2 = plant 2; P3 = plant 3. The values with different letters are significantly different (*p* < 0.05), using one-way ANOVA and multiple pairwise-comparison Tukey’s test.

Concentrations of PC, FC, TC and TTC were variable between the three individuals and between collections (Figure 1). Similar variation patterns were observed in PC and TC (Figure 1a,c), which corresponded to incipient and intermediate phenological stages, with the exception of the later senescence stage. For FC and TTC, an increasing trend was registered in the senescence stages (Figure 1b,d). No significant differences were observed between individuals regarding the total content of flavonoids (FC), tannins (TC) and triterpenoids (TTC), except for PC (*p* < 0.05). For PC and TC, there were significantly high peaks for plant 3, during the fruit-development stage (C3) (Figure 1a,c). The highest PC values were recorded at C3 in plants 2 (491 ± 52.2 mg GAE/g DW, *p* = 0.12) and 3 (547.1 ± 51.6 mg GAE/g DW, *p* < 0.001) and at C1 for the first plant (388.6 ± 101.5 mg GAE/g DW, *p* = 0.09). The highest TC values were observed at C1 for plant 1 (166.5 ± 81.3 mg CE/g DW, *p* = 0.06), at C3 for plant 3 (322.8 ± 2.3 mg CE/g DW, *p* < 0.05) and at C5 for plant 2 (215.8 ± 61 mg CE/g DW, *p* = 0.08). Regarding total flavonoid accumulation, for each plant during the nine collections, significantly increased values were noticed between the flower, fruit, seed and senescence stages (Figure 1b). The highest values were registered at C8 in plants 2 (180.7 ± 7.3 mg RE/g DW, *p* < 0.001) and 3 (135.0 ± 2.8 mg RE/g DW, *p* < 0.001) and at C9 for plant 1 (113 ± 6.2 mg RE/g DW, *p* < 0.001). Significantly high TTC values were registered only during the senescence stages (C8, C9) (Figure 1d) for plant 1 (25.1 ± 1.7 mg CAE/g DW, *p* = 0.08) and plant 2 at C9 (32.5 ± 5 mg CAE/g DW, *p* < 0.001) and for plant 3 at C8 (31.7 ± 1.6 mg CAE/g DW, *p* < 0.001). According to these results, the methanolic extracts of leaves corresponding to plant 2 showed the highest concentrations of total phenols and flavonoids, while plant 3 presented the highest concentrations of tannins and triterpenoids.

### 2.2. LC–MS/MS and HPLC–UV–VIS–DAD Phytochemical Characterization

**Figure 2:**
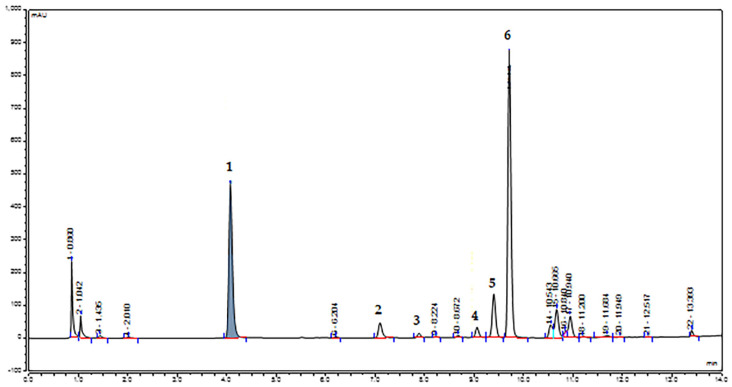
HPLC-UV-VIS-DAD chromatogram at 267 nm of smoketree methanolic extracts. The numbered peaks correspond to the identified and quantified compounds presented in Table 1, identified using LC–MS/MS.

The methanolic extracts corresponding to each plant (P1–P3) and collection (C1–C9) were characterized using LC–MS/MS and HPLC–UV–VIS–DAD (Figure 2). The initial separation was performed using a reversed phase LC–MS/MS method and six major compounds were identified, using spectral data and MS/MS fragmentation patters (Supplementary Figures S2–S7), which were compared with those present in the PubChem database [57] and in previously published papers [40,58,59,60,61,62] (Table 1).

Quantitative determinations using HPLC–UV–VIS–DAD were performed for five of the most abundant compounds: methyl gallate, methyl digallate I, myricetin-3-O-galactoside, myricitrin and pentagalloyl glucose (Table 2, Supplementary Figures S2–S6). Although initially identified using LC–MS/MS, we found only traces of compound 5 (methyl digallate II, Rt = 9.39 min, *m*/*z* = 335) when the quantitative determinations were performed. The content of these compounds varied between individuals and collections. Methanolic extracts from samples of plant 2 presented the highest quantities of these compounds, followed by samples from plant 3 and plant 1, respectively. The concentrations of methyl gallate varied significantly between individuals (*p* < 0.05) and collections for each plant (*p* < 0.001), reaching high values, for all plants, during the flowering stage (C1). Pentagalloyl glucose accumulated differently in the three individuals (*p* < 0.05) and along the nine collections, for each plant. Similar to methyl gallate, this compound registered a peak in plant 1 (*p* < 0.001), plant 2 (*p* = 0.08) and plant 3 (*p* < 0.001) at the first collection, during the flowering stage. Differences in methyl digallate I accumulation were not significant between individuals. This compound reached its peak for plants 1 (*p* < 0.001) and 2 (*p* < 0.001) in C4 and for plant 3 in C3 (*p* < 0.001), during the fruit stages. Myricetin-3-O-galactoside concentrations differed significantly between individuals (*p* < 0.001) and were highest for plant 1 in C5 (*p* < 0.05), plant 2 in C3 (*p* < 0.01) and plant 3 in C8 (*p* < 0.05), while myricitrin was mostly undetected and registered its peak for plant 2 in C9.

### 2.3. Antioxidant Activities (DPPH, FRAP, CUPRAC, TEAC) and Correlations with the Contents of Metabolites

**Figure 3:**
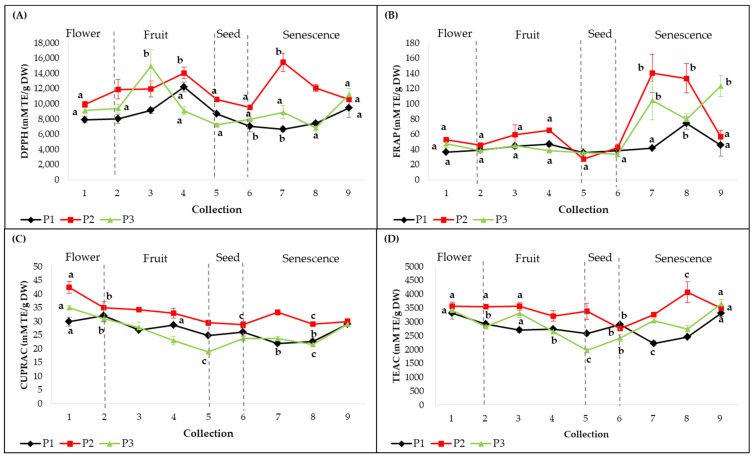
Antioxidant capacities of smoketree methanolic extracts (expressed as mM TE/g DW): (**A**) DPPH, (**B**) FRAP, (**C**) CUPRAC and (**D**) TEAC. P1 = plant 1; P2 = plant 2; P3 = plant 3. The values with different letters are significantly different (*p* < 0.05), using one-way ANOVA and multiple pairwise-comparison Tukey’s test.

Methanolic extracts obtained from leaves collected from all three individuals during the sampling period (C1–C9) were assessed for antioxidant capacity using four in vitro assays (Figure 3). According to the DPPH and CUPRAC assays, the antioxidant activities were significantly different between individuals (*p* < 0.01). The highest antioxidant capacity measured by DPPH was evident at C4 for plant 1 (12.19 ± 0.66 M TE/g DW, *p* < 0.001), at C7 for plant 2 (15.45 ± 1.21 M TE/g DW, *p* < 0.001) and at C3 for plant 3 (14.92 ± 2.219 M TE/g DW, *p* < 0.001) (Figure 3a). The CUPRAC assay revealed that the highest values were recorded at C1 for plants 2 (42.4 ± 2.2 mM TE/g DW, *p* < 0.001) and 3 (34.9 ± 0.7 mM TE/g DW, *p* < 0.001) and C2 for plant 1 (32 ± 2.1 mM TE/g DW, *p* < 0.001) (Figure 3c). These maximum peak activities were significantly higher than the rest of the registered values.

In the FRAP and TEAC assays, there were no significant differences between plants. The best antioxidant activities on Fe^3+^ were registered in advanced stages of senescence, as follows: at C8 for plant 1 (74.5 ± 8.2 mM TE/g DW, *p* < 0.05), at C7 for plant 2 (140.2 ± 25.2 mM TE/g DW, *p* < 0.001) and at C9 in plant 3 (123.3 ± 14 mM TE/g DW, *p* < 0.001) (Figure 3b). The TEAC assay showed a significant difference between individuals (*p* < 0.01) and high antioxidant activities were evident at C9 for plants 1 (3316.4 ± 116.7 mM TE/g DW, *p* < 0.001) and 3 (3661.1 ± 160.5 mM TE/g DW, *p* < 0.001) and at C8 for plant 2 (4074.7 ± 374.6 mM TE/g DW, *p* < 0.01) (Figure 3d). The FRAP and TEAC assays displayed significant increases in the senescence phases. According to these results, the methanolic extract of leaves corresponding to plant 2 showed the highest antioxidant capacity, with variations between the collections and the used methods.

The Pearson’s correlation coefficient revealed that there are only punctual, statistically significant correlations between the individual compound concentrations and the antioxidant activities (Supplementary Table S1). In the second collection (C2), we observed significantly strong correlations between the antioxidant activity assayed using TEAC and the contents of methyl gallate (R^2^ = 0.999, *p* < 0.01) and pentagalloyl glucose (R^2^ = 0.999, *p* < 0.001). In the same collection, the antioxidant activity measured using DPPH was positively correlated with myricetin-3-O-galactoside. In addition, strong correlations were observed between the content of methyl gallate and the antioxidant activity measured using the FRAP assay, in the fourth collection (R^2^ = 0.999, *p* < 0.05) and in later collections (C7) between the content of pentagalloyl glucose (R^2^ = 0.999, *p* < 0.05) and the antioxidant activity using the CUPRAC assay.

### 2.4. Dynamics of Secondary Metabolite Content Depending on the Phenological Phases

The variations of the environmental parameters registered during this study are presented in Figure S1 (Supplementary Material), while details regarding the phenological stages are presented in Section 4.1 (Table 3).

**Figure 4:**
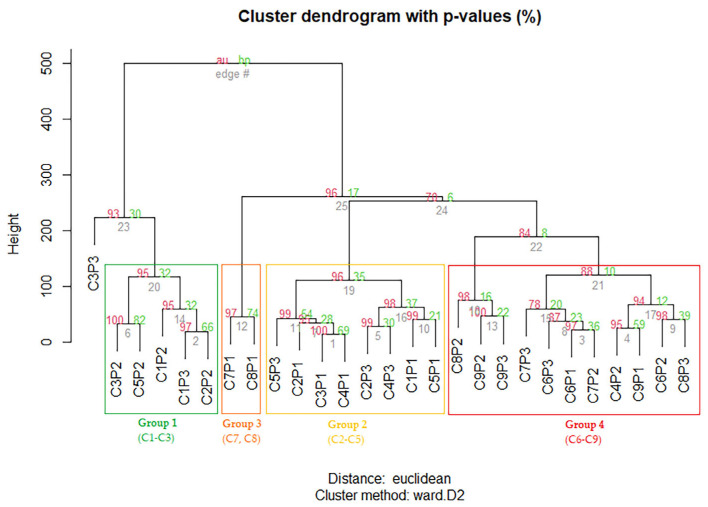
HCAbp based on the metabolic profile of smoketree leaves collected during this study (C1–C9), with samples grouped by similar metabolisms included in differently colored rectangles.

HCAbp (Hierarchical Clustering Analysis with bootstrap resampling) was performed using the quantitative data of major compounds and compound classes and the results showed a clear clustering tendency into four clusters (groups), according to the phenological stages (Figure 4b). In general, we obtained bootstrap values higher than 80%, which support the distinguished clusters. Group 1 mainly consisted of samples collected during the first and third collections (C1 to C3), corresponding to the inflorescence and fruit development stages, except for leaves collected from one individual in an advanced phenological stage, corresponding to seed ripening (C5). The samples clustered under this group generally presented high levels of methyl gallate and pentagalloyl glucose and relatively high levels of PC and TC. The leaves collected from the plant 1 during the senescence stage (C7, C8) clustered into a separate group (Group 3, Figure 4b), while the leaves collected from samples during flowering, fruit development and fruit and seed ripening (C2–C5) formed a separate group (Group 2, Figure 4b). This group measured the highest contents of methyl digallate I and relatively high levels of methyl gallate, pentagalloyl glucose, PC, FC and TC. In addition, it appeared to be more metabolically similar to leaves from plants in their late phenological stages (Group 4, Figure 4b) than to the leaves collected from plants at earlier stages (Group 1). Group 4 presented the highest levels of myricetin-3-O-galactoside, myricitrin, FC and TTC and comprised leaves collected from plants during the early and late senescence stages (C6 to C9), except for leaves collected from one individual during the fruit-ripening stage (C4) (Figure 4b). According to this analysis, plant 1 was metabolically different than the other two plants, displaying prolonged fruit and seed stages. During the initial collections, plant 1 displayed metabolite contents similar to those determined during the early fruit stages in the other two plants, whereas in later collections (C7 and C8) the contents were similar to those described during the later fruit stages and senescence in the other individuals.

### 2.5. Variation of Secondary Metabolite Content under the Influence of the Environmental Factors

**Figure 5:**
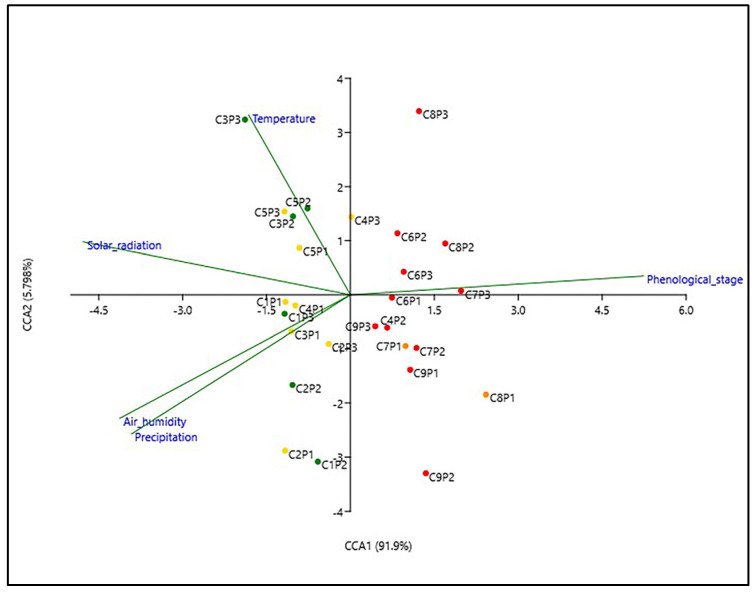
CCA based on the metabolic profile of smoketree leaves collected during this study (C1–C9), correlated to the registered environmental factors and phenological stages. The color of the dots shows the belonging of the samples to the groups with similar metabolism resulting from the HCAbp analysis (group 1—green, group 2—yellow, group 3—orange, group 4—red).

For each collection, the metabolomic data were correlated with the plant phenological stages and environmental variables, by applying CCA (Canonical Correlation Analysis) (Figure 5). According to the results, the environmental factors exerted different effects on the secondary metabolism of smoketree leaves.

Samples clustered under groups 1 and 2 were influenced by the four environmental parameters in different associations and not by the phenological stages. The secondary metabolism of samples collected during C1 and C2 were more influenced by precipitation and humidity and, to a lower degree, by radiation. Starting from C3 until C5, the metabolism of smoketree leaves was more influenced by temperature and radiation. The accumulation of metabolites in samples from groups 3 and 4 was primarily influenced by the phenological stages. Additionally, the metabolism of leaves collected during the early senescence phases (C6) was influenced to a small extent by temperature, while those collected in the advanced senescence phases (C9) were influenced by precipitation. It is evident that in early phenological stages, the plant metabolism is more responsive to the habitat parameters, while during senescence, the metabolism is mostly independent of the action of environmental factors.

## 3. Discussion

This study reports, for the first time, the influence of environment and phenological phases on main secondary metabolites and the variation of antioxidant activities in *Cotinus coggygria* Scop. during the warm season, using diverse analytical techniques. The compounds identified in this study, by LC–MS/MS, are representative for this species and belong to two groups: flavonoids (myricetin-3-O-galactoside, myricitrin) and hydrolysable tannins (methyl gallate, methyl digallate I, methyl digallate II, pentagalloyl glucose).

As expected, tannins represented the main constituents of smoketree leaves, given that the leaves of Anacardiaceae family members are rich in this class of compound [22]. Pentagalloyl glucose and methyl gallate were first reported in smoketree leaves by Westenburg et al. (2000) [33], while methyl digallate was first mentioned by Rendekova et al. (2016) [40]. The richness in gallic acid derivatives, present especially in leaves, has been reported by other authors as well [63,64,65,66]. The flavonoids present in leaves are generally glycosylated derivatives with D-glucose, L-rhamnose and L-arabinose, in the third position of the aglycon, which is usually represented by myricetin, quercetin and kaempferol [40,63,65,66]. The presence of the two flavonoids identified in our study, myricetin-3-O-galactoside and myricetin-3-O-rhamnoside, was confirmed spectroscopically in the ethyl acetate fraction of smoketree leaves extract [65].

We measured the content of four metabolite classes in this species (polyphenols, flavonoids, tannins and triterpenes) and five of the six most abundant secondary metabolites were quantified by HPLC–UV–VIS–DAD.

Regarding PC and TC, the highest values were generally reported in extracts after the first three collections, with superior values usually registered in C3 (the end of June). These results correspond to the end of the flower and fruit development stage and the beginning of the fruit-ripening stage, being associated with the highest levels of solar radiation. It has been reported that plants exposed to high levels of UV-A and UV-B radiation display higher contents of polyphenols (such as chlorogenic acids, tannins), these compounds being known for their antioxidant capacity, neutralizing free radicals and other ROS [4,67].

The PC values registered in our study were comparable or significantly higher than those reported by others. Savikin et al. (2009) [64] measured a phenolic content of 515.5 ± 8.3 mg GAE/g DW in the leaves of plants collected from Serbia during spring and summer, which was comparable to the maximum values obtained by us at the end of June (547.11 ± 51.65 mg GAE/g DW). The smoketree leaves from Turkey collected in June displayed a PC value of 380.2 ± 6.38 mg GAE/g DW [29], while we registered values between 330.5 ± 33.7 and 452.4 ± 77.9 mg GAE/g DW for samples collected during the same month. Hashoum et al. (2017) [68] monitored the accumulation of total polyphenols in smoketree leaves from France, corresponding to three different seasons (summer, green leaves; fall, senescent leaves; winter, litter leaves) and showed the highest PC value was registered in extracts of senescent leaves (~226 mg GAE/g DW), unlike our determinations that recorded maximum values in the green leaves collected during the fruit stages (C3–C5). Gavinet et al. (2019) [69] evaluated the influence of the phenolic compounds of smoketree leaves (green, senescent and litter) on forest diversity in France. They discovered that senescent leaves were the richest in total polyphenols (~350 mg GAE/g DW). In our study, the concentrations varied between 226.9 ± 7.7 and 421 ± 2.6 mg GAE/g DW during the senescence stages. For tannins, Savikin et al. (2009) [64] reported a content of 13.7 ± 0.9% and 18.5 ± 1% of total tannins in extracts derived from flowers and leaves, respectively, while Buziashvili et al. (1973) [63] reported a maximum of 18–20% tannins in leaf extracts, during the flowering period. These values are comparable or lower than the ones reported in our study.

Contrary to other studies reporting higher flavonoid contents in plants exposed to increased solar radiation levels [4,12], we noticed an increasing trend in flavonoid accumulation beginning with C4 (the middle of July), with highest values in C8–C9 (September), in advanced phases of senescence. These phases corresponded to early and advanced senescence stages and were associated with decreases in temperature, precipitation and solar radiation levels. A possible explanation for the increasing flavonoid accumulation in the later phenological stages could be their involvement in several cellular processes, such as defense, reproduction (seed dispersal), senescence and even apoptosis [70]. In addition, we can speculate that the sudden increase was triggered by the high amount of solar radiation registered at C3, combined with the rise in temperature between C3 and C4, with these variables being considered crucial in flavonoid bioaccumulation [71]. Our flavonoid content determinations for the samples collected during June varied between 22.5 ± 1.9 and 61.3 ± 7.7 mg RE/g DW, while extracts from the Turkish leaves measured 68.4 ± 1.5 mg CE/g DW [29]. The values measured in extracts from the senescent leaves collected in our study were four times higher (180.7 ± 7.3 mg RE/g DW) than those reported by Gavinet et al. (2019) [69] in the senescent leaves of smoketree from France (~45 mg quercetin equivalents/g DW).

Triterpenoids are a group of highly diverse compounds, involved in signaling and defense [72] and, in this study, the TTC accumulation trend increased in the senescent stages, but not as significantly as in the case of flavonoids. We have not found other reports on the content of total triterpenoids in smoketree, but several studies present the chemical profile of smoketree essential oils [23,25,26,41,42,43,44,45].

Plants’ metabolism is highly sensitive to environmental changes and can be chemically distinctive between individuals of the same species growing in different locations [12]. In this study, the secondary metabolism of the plant material originated in Romania (the Dobrogea region) presented a relative heterogeneity in terms of chemical composition. Myricetin-3-O-galactoside and myricitrin were found in low quantities, with the latter being mostly undetected. However, we measured their highest amounts in the advanced phenological stages, while methyl gallate and pentagalloyl glucose registered their maximum values in the flowering stage (C1 and C2) and methyl digallate I during the fruit development (C3) and ripening stages (C4) (Table 2). The smoketree stems collected between May and June, from Southern Serbia, contained 511.5 ± 0.5 µg/g dry extract of myricetin [35]. Rendekova et al. (2015) [40] reported the contents of methyl gallate (31.2 ± 0.03 µg/g dry extract) and myricetin rhamnoside (8.1 ± 0.09 µg/g dry extract) in a powdered leaf extract from Bulgaria, while we report a maximum concentration of 3.4 ± 0.1 mg RE/g DW of myricitrin and 1.1 ± 0.1 mg RE/g DW of myricetin-3-O-galactoside. The extracts of smoketree aerial parts (shoots/leaves/flowers in a 4:2:1 ratio) growing in the Kaliningrad region contained approximately 3.51 (±0.1) mg/kg methyl gallate and 21.14 (±0.63) mg/g pentagalloyl glucose [27], while a distinct accumulation of these compounds, according to regional provenance, was reported by Sukhikh et al. (2021b) [28]. Here, samples harvested from the Eastern Baltics (Russia) accumulated more quantities of methyl gallate (2.98 ± 0.08 mg/kg) and pentagalloyl glucose (17.64 ± 0.52 mg/kg) than samples from the Moscow region (2.25 ± 0.08 and 15.38 ± 0.52 mg/kg, respectively) and the Minsk region in Belarus (2.71 ± 0.08 and 16.45 ± 0.52 mg/kg, respectively) [28]. These concentrations are comparable to those determined in our study for pentagalloyl glucose (1.9 ± 0.1–20.6 ± 2.7 mg GAE/g DW) and methyl gallate (0.5 ± 0.0–21.4 ± 0.4 mg GAE/g DW). Although precise environmental indicators were not stated by any authors of the previously mentioned studies, the registered variations could be explained by different geographical locations, habitat variables and plant genotypes, which may affect the secondary metabolites accumulation in this species.

To better understand the antioxidant potential of a plant extract, it is necessary to use complementary antioxidant assays. Given the specificity and limitations of each method, we tested the antioxidant activity of the leaf extracts using four different assays, with interesting outcomes (Figure 3a–d). Our extracts from the smoketree leaves collected in this study, at different phenological stages, showed the highest affinity towards the synthetic radical DPPH, followed by the ABTS ion, Fe^3+^–TPTZ and Cu^2+^–neocuproine. In contrast, Sukhikh et al. (2021) [27] reported that the ABTS assays displayed the best radical scavenging capacity, followed by the DPPH, TEAC and FRAP assays; however, our results are several orders of magnitude higher than those reported in their study. The variation patterns of the antioxidant activity during the 4.5 months of monitoring are different from one individual to another, however some trends can be observed. Thus, an increased affinity for the Fe^3+^ ion in the senescence stages (C7–C9) was highlighted. In addition, similar patterns of variation were observed for the antioxidant activity determined by the CUPRAC assay and the accumulation of the two major metabolites, pentagalloyl glucose and methyl gallate, with maximum values observed in the flowering stages (C1–C2) and a tendency to decrease with senescence. 

After conducting a correlation test between the antioxidant activities and the quantified individual compounds, we found few statistically significant correlations. However, a good correlation was observed between some compound concentrations and antioxidant activity determined by all methods, in certain phenological stages. Thus, pentagalloyl glucose and methyl gallate contributed to the total antioxidant activity (DPPH, FRAP, CUPRAC, TEAC) in the C2 and C4 stages, as shown by the correlation coefficient (R^2^) varying between 0.888 and 0.999 (Supplementary Table S1). In addition, the concentration of methyl digallate I was related to the total antioxidant activity in C8 (R^2^ = 0.889–0.995), while myricetin 3-O-galactoside had an influence in C1 and C7 (R^2^ = 0.981–0.996). In our study, the antioxidant activity of leaf extracts was due to several compounds as shown by preliminary HP–TLC investigations, where almost all the separated bands presented antioxidant activity (Supplementary Figure S8). In addition, all major compounds identified in our extracts using LC–MS/MS have been previously reported to be potent antioxidants [46,52,73]. 

In this work, we assessed the impact of one intrinsic (plant phenological stage) and four extrinsic factors (temperature, air humidity, precipitation, solar radiation) on the secondary metabolism of smoketree leaves. Although the individuals belonged to the same population and were exposed to the same environmental conditions, the metabolism of each plant responded differently, thus pointing out the possibility that there were several other factors that regulated the metabolism of smoketree (e.g., plant age, genetic inheritance, biotic factors, sun exposition). Furthermore, more complex studies considering a greater number of factors are needed to advance the knowledge regarding the plasticity of the secondary metabolism of this species.

## 4. Materials and Methods

### 4.1. Plant Material

In this study, *Cotinus coggygria* Scop. plants were selected from a site (44°82′88″ N, 28°75′44″ E) administered by the Babadag Forestry Division (Babadag, Tulcea County, Romania). A voucher specimen (ID: BUCA161.396) was deposited at the BUCA Herbarium of the Institute of Biology Bucharest of Romanian Academy, Bucharest, Romania.

The sampling of smoketree leaves began at the end of May, after full formation of young leaves and continued every 15 days (±2) until the end of September, for a total period of 4.5 months (Table 3). Three healthy, adult individuals (~1.7 m height) were selected, situated approximately 10 m apart from each other. Each collection was performed at the same time of the day (17:00–18:00), with the leaves corresponding to branches most exposed to sun being sampled. The sampled leaves were placed in plastic bags and transported under cooling conditions and finally stored at −20 °C until analyzed. For each timepoint, the phenological stage was recorded and details are presented in Table 3.

**Table 3:** The phenological stages of smoketree plants registered during the sampling period (May–September 2021).

Phenological Stage	Collection	Month(s)
1. Full leaf and inflorescence emergence	C1	End of May
2. Flowering	C2	Mid-June
3. Fruit development	C3	End of June
4. Fruit ripening	C4	Mid-July
5. Seed ripening	C5	End of July
6. Senescence (early)	C6 C7	Mid-August End of August
7. Senescence (advanced)	C8 C9	Mid-September End of September

### 4.2. Environmental Data

The habitat variables used in this study included temperature (°C), precipitation (mm), humidity (%) and solar radiation (Wh/m^2^). For each timepoint, the data corresponding to temperature, rainfall and humidity were obtained from the National Meteorology Agency (ANM), https://www.meteoromania.ro/ (accessed on 1 October 2021), while data on solar radiation were obtained from Copernicus, https://www.copernicus.eu/ (accessed on 2 October 2021). For the data analysis, the average values of temperature, precipitation and humidity registered in the interval between two collections were considered, while in the case of solar radiation, we used the accumulated value registered for the entire collection day. These data were further used for correlation analysis, after appropriate transformation.

### 4.3. Chemicals

The organic solvents used for mobile phases and extractions (methanol, acetonitrile) were of LC–MS purity and were acquired from Carl Roth (Carl Roth, Germany). The ultrapure water and MS-grade formic acid was manufactured by Fisher Chemical (Thermo Fisher Scientific, Waltham, MA, USA). For the colorimetric determinations (Folin-Ciocâlteu’s phenol reagent, Na_2_CO_3_, NaNO_2_, AlCl_3_, NaOH, vanillin, glacial acetic acid, perchloric acid, HCl, PVP) and antioxidant assays (DPPH, Trolox, CH_3_COONa, TPTZ, FeCl_3_, CH_3_CO_2_NH_4_, CuCl_2_, neocuproine, Na_2_S_2_O_8_, ABTS), the substances were purchased from Sigma (Merck, Germany), while the HPLC-grade standards (gallic acid, rutin, corosolic acid, catechin) were acquired from Extrasynthese (Genay, France).

### 4.4. Extract Preparation

For extract preparation, 1 g of smoketree leaves (fresh weight) corresponding to each plant and collection were crushed using a mortar and pestle, then 10 mL of methanol were added, and the samples were vigorously vortexed for 1 min at room temperature (RT). The homogenized samples were placed on a Heidolph Unimax 1010 shaker (Heidolph Instruments GmbH, Germany) and extracted for 2 days under continuous shaking (300 rpm, RT) at room temperature. After this, samples were centrifuged (5 min, 12,000 rpm, 4 °C) using a Universal 320R centrifuge (Hettich, Germany). The supernatant was transferred into a clean tube, the final extract being filtered using a 0.2 µm PTFE filter and stored at −20 °C until needed.

### 4.5. LC–MS/MS and HPLC–UV–VIS–DAD Analysis

For the initial characterization, 1 mL of each extract was pooled to create a representative sample and the investigations were carried out on a Varian LC instrument coupled to a Varian 310 triple quadrupole mass spectrometer (MS) and an electrospray interface (ESI) (Varian, Palo Alto, CA, USA). The mass spectrometer was tuned using a PPG (polypropylene glycol) standard for both the positive and negative mode. For the separation, a Gemini^®^ NX-C18 column (100 × 4.6 mm, 110 Å, 3 μm) from Phenomenex (Torrance, CA, USA) was used. Acidified double distilled water (0.02% formic acid, *v*/*v*) and a solution of acetonitrile:methanol (1:1, *v*/*v*) were used as mobile phases A and B, respectively. The column was initialy equilibrated for 10 min using both the mobile phases (A:B = 90:10). The following gradient elution and flow rates were used: 0 to 20 min: 10–30% B (0.6–0.8 mL/min); 20:01 to 22 min: 100% B (0.8 mL/min); 22:01 to 30 min: 10% B (0.8–0.6 mL/min). The injection volume was 3 μL and the column temperature was set at 40 °C throughout the experiments. For analysis, the following parameters were applied: cappilary voltage: +5.0 kV and −4.5 kV for positive and negative modes, respectively; drying gas pressure: 1.25 atm; drying gas temperature: 300 °C; nebulising gas pressure (N_2_): 3.75 atm. The spectral data were acquired in the range of 100 to 1000 *m*/*z*. Compound assignations were carried out using spectral data and fragmentation patterns available in the PubChem database [57] and in other scientific papers [40,58,59,60,61,62].

After compound identifications, reconfirmation and quantitative determinations were performed using a Vanquish HPLC, coupled to a VF-P32-A pump and a VF-D11-A UV–VIS–DAD detector (Thermo Fisher Scientific, Waltham, MA, USA), using a method adapted after several studies investigating the secondary metabolism of other Anacardiaceae family members [58,62]. A volume of 0.2 µL diluted extract (10-fold dilution) was injected twice, corresponding to each individual and collection. Acidified double-distilled water (phosphoric acid, pH 2), methanol and acetonitrile were used as mobile phases A, B and C, respectively, using the following gradient elution and flow rates: 0 to 9:59 min: 5% B/5% C (0.3 mL/min); 10 min: 15% B/15% C (0.5 mL/min); 14 min: 50%B/15% C (0.6 mL/min). The chromatograms were acquired at 267 nm and UV–VIS–DAD spectra were recorded in the range of 200–800 nm. In the case of hydrolysable tannins, the UV spectra of the components derived from gallic acid were identical, therefore the chromophore group was considered to be exclusively given by gallic acid. 

### 4.6. Quantitative Determinations

HPLC–UV–VIS-DAD quantifications were carried out by calculating the response factor of a solution of gallic acid (purity 97%), for hydrolysable tannins and rutin (purity 98%) for flavonoids, of known concentrations. The following formula was used: A = B/C × D × E/F, where A = the concentration of the investigated compound; B = the peak area of the investigated compound; C = the peak area of gallic acid (14.612 mAU*min) or rutin (1.322 mAU*min); D = the concentration of gallic acid (238.6 µg/mL) or rutin (22.5 µg/mL); E = the dilution factor (5); and F = sample/reference injection ratio (2.5).

All colorimetric and antioxidant measurements were performed on a Spectronic Helios Gamma UV–VIS spectrophotometer (Thermo Fisher Scientific), in biological and technical triplicate, after appropriately diluting the crude extract. For the dry weight estimation, 1 g of leaves corresponding to each plant and each collection was dried in the oven (5 h, 60 °C) and finally weighed. The difference between the final and initial weight represented the dry weight (DW) per g of fresh weight.

#### 4.6.1. Evaluation of Total Phenolic Content (PC)

The total phenolic content was determined using the Folin–Ciocalteu method [74]. The results were expressed as mg of gallic acid equivalents (GAE)/g of DW, based on the gallic acid calibration curve (final concentration: 10–100 µg/mL, R^2^ = 0.9968).

#### 4.6.2. Evaluation of Total Flavonoid Content (FC)

The total flavonoid content was measured using a protocol adapted from Cai et al. (2010) [75]. The results were expressed as mg of rutin equivalents (RE)/g of DW, based on the rutin calibration curve (final concentration: 100–1000 µg/mL, R^2^ = 0.9996).

#### 4.6.3. Evaluation of Total Triterpenoid Content (TTC)

The content of total triterpenoids was determined according to Ke et al. (2014) [76]. The results were expressed as milligrams of corosolic acid equivalents (CAE)/g of DW, based on the corosolic acid calibration curve (final concentration: 10–50 µg/mL, R^2^ = 0.9769).

#### 4.6.4. Evaluation of Total Tannin Content (TC)

The content of total tannins was measured using an adapted protocol from Makkar et al. (1993) [77]. The TC concentration was calculated as the difference between the phenol concentrations before and after PVP precipitation. The results were expressed as mg of catechin equivalents (CE)/g of DW, based on the catechin calibration curve (final concentration: 60–140 µg/mL, R^2^ = 0.9976). 

#### 4.6.5. Evaluation of Antioxidant Activity through DPPH, FRAP, CUPRAC and TEAC Assays

The DPPH (2,2-Diphenyl-1-picrylhydrazyl) assay was applied according to Marxen et al. (2007) [78]. The results were expressed as mM of Trolox equivalents (TE)/g of DW, according to a Trolox standard curve (final concentration: 50–150 µg/mL, R^2^ = 0.9873).

The FRAP (Ferric-Reducing Antioxidant Power), CUPRAC (Cupric-Reducing Antioxidant Power) and TEAC (Trolox Equivalent Antioxidant Assay) assays were conducted according to the protocols described by Chamorro et al. (2019) [79].

For FRAP, results were expressed as mM of Trolox equivalents (TE)/g of DW, according to a Trolox standard curve (final concentration: 0.2–1 mM, R^2^ = 0.9979).

For CUPRAC, results were expressed as mM of Trolox equivalents (TE)/g of DW, according to a Trolox standard curve (final concentration: 0.25–2 mM, R^2^ = 0.9997).

For TEAC, results were expressed as mM of Trolox equivalents (TE)/g of DW, according to a Trolox standard curve (final concentration: 25–250 µg/mL, R^2^ = 0.9935).

#### 4.6.6. Evaluation of Antioxidant Activity by HP–TLC

The methanolic leaf extracts were separated using HP–TLC performed according to Ramírez-Briones et al. (2017) [80]. The metabolic fingerprints were visualized under UV light (254 nm). For the antioxidant activity evaluation, the plate was sprayed with a 0.2% methanolic DDPH solution and visualized under visible light, using a CAMAG TLC visualizer device (CAMAG, Muttenz, Switzerland).

### 4.7. Statistical Analysis

The data obtained in this study were subjected to multivariate analysis using MS Excel, R 3.0.3 (R Foundation for Statistical Computing, Austria) [81] and PAST 4.03 [82]. The values corresponding to compound classes, individual compounds and antioxidant activities were subjected to the one-way Analysis of Variance (ANOVA) for statistical significance assessment, followed by Tukey’s HSD test to determine the differences between means. The values registered for the environmental variables were transformed using the arcsin method (for percentage values) and the log method (for the other categories). We used the R program to perform HCAbp, using the packages *pvclust* and *factoextra*. To correlate the metabolomic data with the habitat variables, we applied CCA, using the package *vegan*. The strength of the correlation between individual metabolites and antioxidant activities was assessed using the Pearson correlation, performed in R. The error bars of the graphics represent the standard error of mean value (±SE) and *p* values *p* < 0.05 (*), *p* < 0.01 (**) and *p* < 0.001 (***) were considered statistically significant. PAST, R and MS Excel were used for the graphical representations.

## 5. Conclusions

In the present study, the secondary metabolism of three *C. coggygria* Scop. individuals was characterized, in correlation with several environmental factors. The accumulation of polyphenols and tannins presented several peaks in the fruit development stages, while higher contents of flavonoids and triterpenes were determined during senescence.

Five compounds were identified and quantified, belonging to two groups: flavonoids (myricetin-3-O-galactoside, myricitrin) and hydrolysable tannins (pentagalloyl glucose, methyl gallate, methyl digallate I). Flavonoid compounds were more abundant in the later phenological phases, methyl gallate and pentagalloyl glucose were highest in the flowering stage and methyl digallate I was synthesized in high quantities during the fruit stages. The metabolism of leaves collected during the flower, fruit and seed stages was influenced by solar radiation, precipitation and temperature, or a combination of these environmental factors registered in the habitat. In the case of samples collected during the senescence stages, the secondary metabolism evolved according to the phenological stage of the plants.

## Figures and Tables

**Table 1:** Major compounds identified in smoketree methanolic extracts.

Peak No.	Rt (Min)	Tentative Identification	[M-H]^−^ (*m*/*z*)	λ_max_ (nm)	Formula	Major Fragments	References
1	4.06	Methyl gallate	183	275	C_8_H_8_O_5_	124, 168	[40,59,61,62], PubChem
2	7.09	Methyl digallate I	335	285	C_15_H_12_O_9_	183	[40,59,61,62]
3	7.88	Myricetin 3-O-galactoside	479	267, 305, 365	C_21_H_20_O_13_	316, 270	[60,61,62], PubChem
4	9.05	Myricetin-3-O-rhamnoside (Myricitrin)	463	265, 303, 356	C_21_H_20_O_12_	316, 286, 107	[40,58,60,61], PubChem
5	9.39	Methyl digallate II	335	276	C_15_H_12_O_9_	183	[40,59,61,62]
6	9.70	Pentagalloyl glucose	939	284	C_41_H_32_O_26_	769, 787, 617, 261, 294, 429	[40,60,61,62]

**Table 2:** The content of major secondary metabolites identified in smoketree methanolic extracts (tannins expressed as mg GAE/g DW and flavonoids expressed as mg RE/g DW).

Compounds	Pn/Cn	Flowering Stages		Fruit and Seeds Stages			Senescence Stages			
		C1	C2	C3	C4	C5	C6	C7	C8	C9
Myricetin- 3-O-galactoside	P1	0.1 ± 0.0 ^a^	0.1 ± 0.0 ^ab^	0.1 ± 0.0 ^a^	0.1 ± 0.0 ^b^	0.2 ± 0.0 ^b^	0.1 ± 0.0 ^ab^	0.1 ± 0.0 ^a^	0.1 ± 0.0 ^a^	0.2 ± 0.0 ^c^
	P2	0.8 ± 0.0 ^a^	0.9 ± 0.0 ^ab^	1.1 ± 0.1 ^b^	0.9 ± 0.0 ^ab^	1.0 ± 0.0 ^ab^	1.0 ± 0.0 ^ab^	0.9 ± 0.0 ^a^	1.0 ± 0.0 ^ab^	0.8 ± 0.0 ^a^
	P3	0.5 ± 0.0 ^a^	0.5 ± 0.0 ^a^	0.5 ± 0.0 ^a^	0.4 ± 0.0 ^a^	0.4 ± 0.0 ^a^	0.3 ± 0.0 ^b^	0.4 ± 0.0 ^a^	0.6 ± 0.0 ^c^	0.4 ± 0.0 ^a^
Myricitrin	P1	ND	0.03 ^a^	ND	ND	ND	ND	ND	0.08 ^b^	ND
	P2	ND	0.2 ± 0.1 ^a^	ND	0.08	ND	ND	0.2 ± 0.0 ^a^	ND	3.4 ± 0.1 ^b^
	P3	0.07	ND	ND	ND	ND	ND	ND	ND	ND
Methyl gallate	P1	16.9 ± 0.3 ^a^	12.6 ± 0.4 ^b^	13.5 ± 0.5 ^ab^	7.3 ± 0.1 ^c^	12.6 ± 0.1 ^b^	10.6 ± 0.0 ^bc^	8.4 ± 0.0 ^bc^	11.9 ± 0.0 ^b^	14.1 ± 0.1 ^ab^
	P2	21.4 ± 0.4 ^a^	16.3 ± 0.5 ^b^	5.3 ± 0.2 ^cd^	15.8 ± 0.1 ^b^	13.5 ± 0.2 ^bc^	13.3 ± 0.3 ^bc^	14.3 ± 0.2 ^bc^	10.8 ± 0.2 ^c^	4.2 ± 0.2 ^d^
	P3	12.3 ± 0.1 ^a^	12.0 ± 0.1 ^ab^	11.7 ± 0.1 ^b^	2.99 ± 0.0 ^c^	5.4 ± 0.1 ^bc^	5.1 ± 0.0 ^bc^	8.4 ± 0.1 ^b^	0.5 ± 0.0 ^d^	10.8 ± 0.0 ^ab^
Methyl digallate I	P1	3.2 ± 0.0 ^a^	3.5 ± 0.1 ^ab^	2.4 ± 0.1 ^ac^	6.3 ± 0.0 ^b^	2.7 ± 0.0 ^ac^	3.9 ± 0.1 ^bc^	2.6 ± 0.0 ^ac^	1.2 ± 0.0 ^c^	2.4 ± 0.0 ^ac^
	P2	2.4 ± 0.3 ^a^	5.0 ± 0.2 ^bc^	7.7 ± 0.5 ^c^	4.2 ± 0.0 ^ab^	4.4 ± 0.0 ^b^	3.9 ± 0.0 ^b^	2.9 ± 0.0 ^ab^	7.7 ± 0.0 ^c^	4.7 ± 0.0 ^b^
	P3	4.9 ± 0.1 ^bc^	4.4 ± 0.5 ^bc^	5.5 ± 0.1 ^c^	4.5 ± 0.0 ^bc^	3.7 ± 0.0 ^b^	5.2 ± 0.2 ^bc^	3.4 ± 0.0 ^b^	1.0 ± 0.0 ^a^	4.1 ± 0.1 ^b^
Pentagalloyl glucose	P1	18.4 ± 0.1 ^a^	15.2 ± 0.7 ^ab^	14.8 ± 0.7 ^ab^	11.9 ± 0.0 ^bc^	14.6 ± 0.0 ^ab^	13.1 ± 0.0 ^b^	9.1 ± 0.0 ^c^	11.7 ± 0.0 ^bc^	15.3 ± 0.0 ^ab^
	P2	20.6 ± 2.7 ^a^	19.6 ± 1.0 ^a^	11.5 ± 0.9 ^bc^	18.0 ± 0.3 ^ab^	16.1 ± 0.3 ^b^	15.0 ± 0.2 ^bc^	14.9 ± 0.4 ^bc^	14.5 ± 0.1 ^bc^	7.8 ± 0.1 ^c^
	P3	15.4 ± 0.1 ^a^	14.6 ± 0.3 ^ab^	15.8 ± 0.3 ^a^	7.2 ± 0.1 ^bc^	7.7 ± 0.0 ^bc^	8.7 ± 0.4 ^b^	10.1 ± 0.0 ^b^	1.9 ± 0.1 ^c^	13.8 ± 0.3 ^ab^

Data are presented as mean ± SD (technical duplicates). Pn, Plant (1, 2, 3); Cn, Collection (1, 2, 3 etc.); DW, Dry Weight; ND, not detected; GAE, Gallic Acid Equivalents; RE, Rutin Equivalents. The values with different letters are significantly different (*p* < 0.05), using one-way ANOVA and multiple pairwise-comparison Tukey’s test.

## Data Availability

All data used and obtained during this study are included in this research paper as figures, tables and Supplementary Files.

## Supplementary Materials

The following supporting information can be downloaded, as a single file, at: https://www.mdpi.com/article/10.3390/plants12091762/s1, Figure S1: Climatograms presenting the variation of the environmental variables; Table S1: Pearson’s correlation coefficient for the content of secondary metabolites and antioxidant activities; Figure S2: Fragmentation pattern of methyl gallate; Figure S3: Fragmentation pattern of methyl digallate I; Figure S4: Fragmentation pattern of myricetin-3-O-galactoside; Figure S5: Fragmentation pattern of myricitrin; Figure S6: Fragmentation pattern of pentagalloyl glucose; Figure S7: Fragmentation pattern of methyl digallate II; Figure S8: HP-TLC of smoketree methanolic extracts.
