# Supplementary material for: Phenological and Environmental Factors’ Impact on Secondary Metabolites in Medicinal Plant Cotinus coggygria Scop
Source: Plants (Basel). 2023 Apr 25;12(9):1762. doi: 10.3390/plants12091762 (PMC10181090; doi:10.3390/plants12091762)
Supplement: Supplementary file 1 [file plants-12-01762-s001.zip › Updated Supplementary File-plants-2305854.pdf]

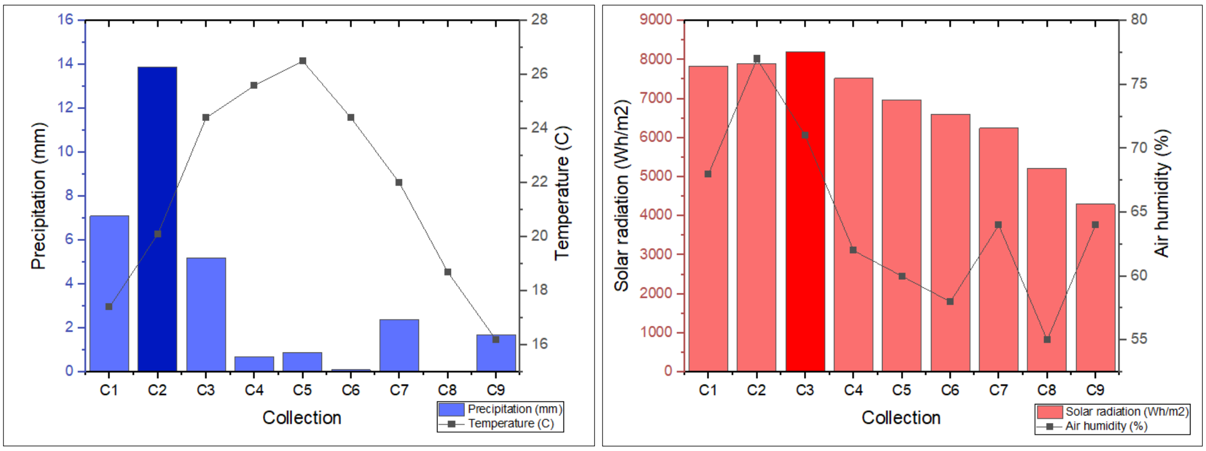

(a) (b)

Figure S1. Climatograms presenting the variation of (a) temperature (°C) and precipitation (mm) and (b) air humidity (%) and solar radiation (Wh/m²) for the nine collections of *Cotinus coggygria* leaves. Darker shades correspond to the highest values registered for the specific variable. Data used for visualization were retrieved from ANM and Copernicus, as discussed in Section 4.2.

Table S1. Pearson's correlation coefficient for the content of secondary metabolites and antioxidant activities in *C. coggygria* methanolic extracts (expressed as R^2).

Collection	Compound	DPPH	FRAP	CUPRAC	TEAC
**C1**	Myricetin-3-O-galactoside	0.995	0.982	0.987	0.983
	Methyl gallate	0.365	0.273	0.585	0.602
	Methyl digallate I	-0.172	-0.076	-0.412	-0.431
	Pentagalloyl glucose	0.294	0.200	0.523	0.540
**C2**	Myricetin-3-O-galactoside	0.994	0.881	0.752	0.840
	Methyl gallate	0.888	0.996	0.990	**0.999****
	Methyl digallate I	0.956	0.774	0.611	0.720
	Pentagalloyl glucose	0.890	0.996	0.989	**0.999*****
**C3**	Myricetin-3-O-galactoside	0.354	0.941	0.964	0.926
	Methyl gallate	-0.191	-0.985	-0.995	-0.849
	Methyl digallate I	0.552	0.844	0.882	0.986
	Pentagalloyl glucose	0.235	-0.967	-0.945	-0.552
**C4**	Myricetin-3-O-galactoside	0.466	0.753	0.522	0.854
	Methyl gallate	0.946	**0.999***	0.965	0.978
	Methyl digallate I	0.009	-0.350	-0.055	-0.504
	Pentagalloyl glucose	0.977	0.988	0.989	0.947

**C5**	Myricetin-3-O-galactoside	0.739	-0.960	0.627	0.737
	Methyl gallate	0.876	-0.575	0.940	0.878
	Methyl digallate I	0.446	-0.794	0.303	0.443
	Pentagalloyl glucose	0.904	-0.625	0.959	0.906
**C6**	Myricetin-3-O-galactoside	0.988	0.700	0.753	0.005
	Methyl gallate	0.466	0.990	0.976	0.806
	Methyl digallate I	-0.189	-0.908	-0.872	-0.943
	Pentagalloyl glucose	0.451	0.988	0.973	0.816
**C7**	Myricetin-3-O-galactoside	0.991	0.956	0.972	0.889
	Methyl gallate	0.970	0.781	0.990	0.659
	Methyl digallate I	0.072	0.481	-0.030	0.629
	Pentagalloyl glucose	0.996	0.870	**0.999***	0.0769
**C8**	Myricetin-3-O-galactoside	0.815	0.908	0.789	0.941
	Methyl gallate	0.519	0.351	0.556	0.268
	Methyl digallate I	0.996	0.994	0.992	0.981
	Pentagalloyl glucose	0.749	0.612	0.777	0.541
**C9**	Myricetin-3-O-galactoside	0.543	0.016	0.893	0.403
	Methyl gallate	-0.475	0.063	-0.926	-0.329
	Methyl digallate I	0.826	0.402	0.649	0.726
	Pentagalloyl glucose	-0.353	0.195	-0.968	-0.200

Values in bold are statistically significant at * $p < 0.05$, ** $p < 0.01$, *** $p < 0.001$, according to Pearson's correlation coefficient.

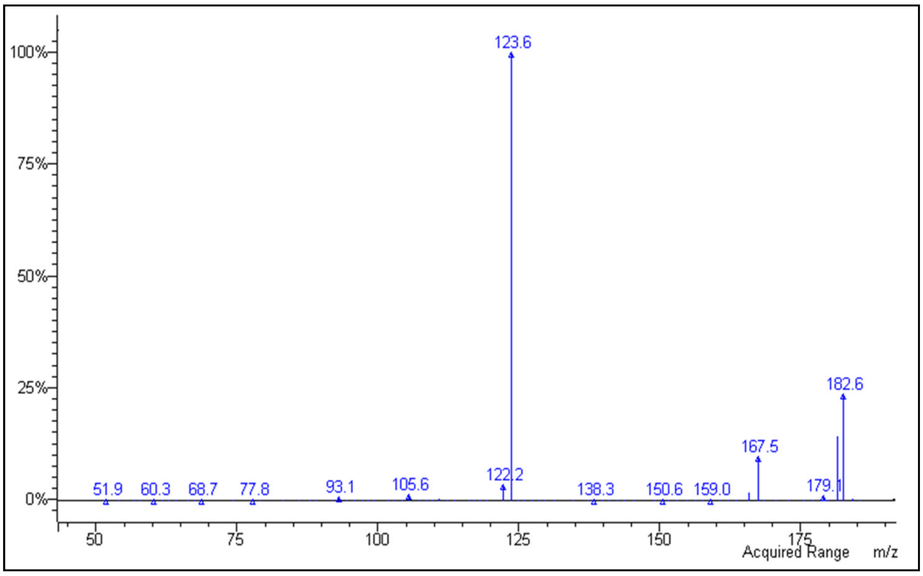

Figure S2. Fragmentation pattern of methyl gallate (m/z = 183) at -20 eV

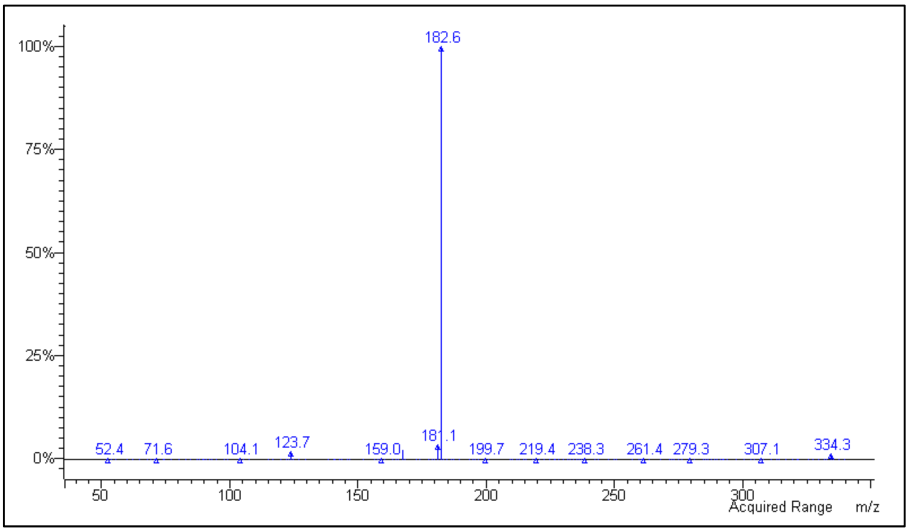

Figure S3. Fragmentation pattern of methyl digallate I (*m/z* = 335) at -20 eV

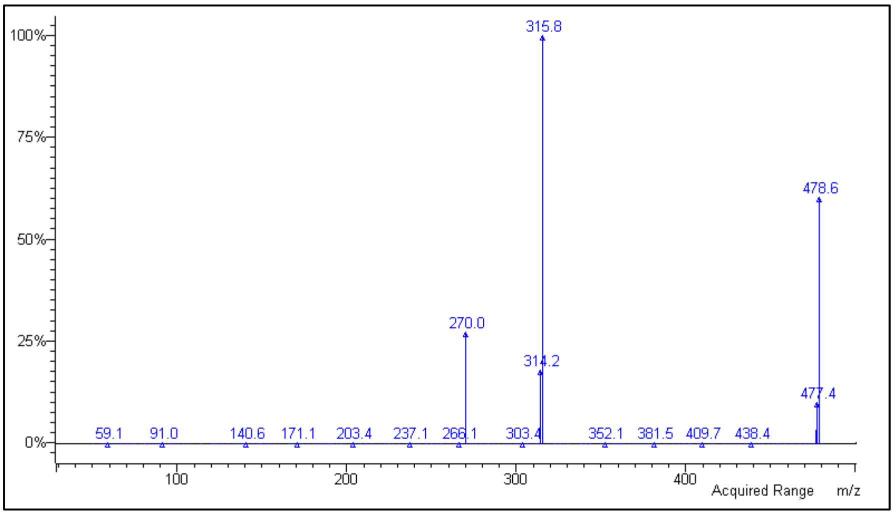

Figure S4. Fragmentation pattern of myricetin-3-O-galactoside (*m/z* = 479) at -25 eV

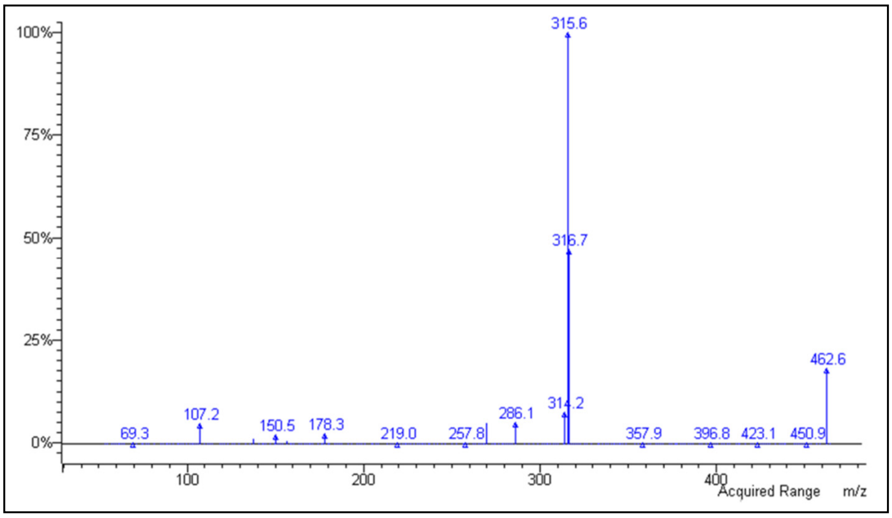

Figure S5. Fragmentation pattern of myricitrin (*m/z* = 463) at -30 eV

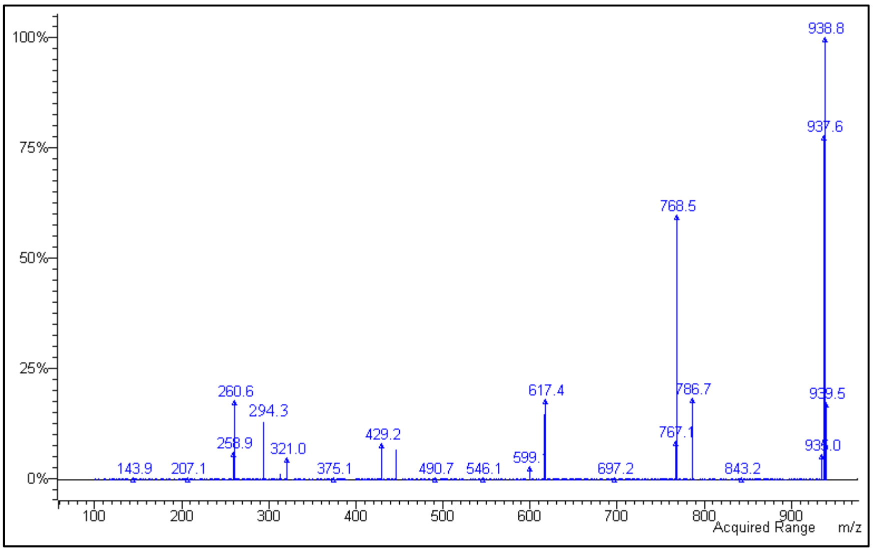

Figure S6. Fragmentation pattern of pentagalloyl glucose (*m*/*z* = 939) at -30 eV

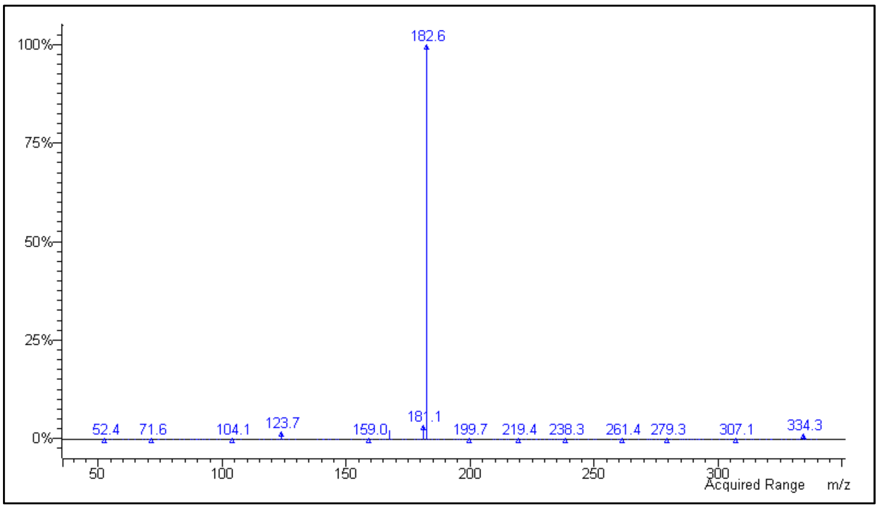

Figure S7. Fragmentation pattern of methyl digallate II (*m*/*z* = 335) at -20 eV

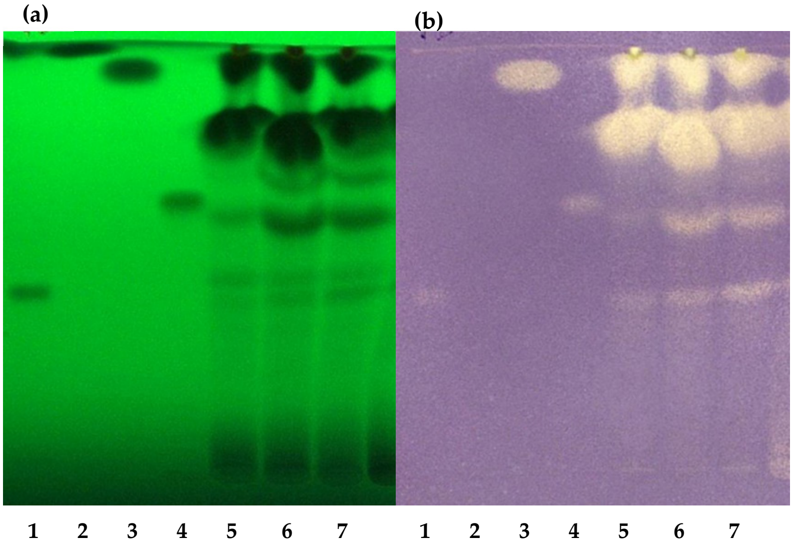

Figure S8. HP-TLC of smoketree methanolic extracts visualized under **a)** UV light (254 nm) and **b)** visible light using DPPH reagent for antioxidant activity testing. Lanes: **1** – rutin; **2** – transcinnamic acid; **3** – gallic acid; **4** – myricetin-3-O-glucoside; **5** – C3P1 sample; **6** – C4P2 sample; **7** – C6P2 sample.
